# H_2_O_2_ rejuvenation-mediated synthesis of stable mixed-morphology Ag_3_PO_4_ photocatalysts

**DOI:** 10.1016/j.heliyon.2018.e00599

**Published:** 2018-04-13

**Authors:** Henry Agbe, Nadeem Raza, David Dodoo-Arhin, Aditya Chauhan, Ramachandran Vasant Kumar

**Affiliations:** a Department of Materials Science & Engineering, University of Ghana, P. O. Box LG 77, Legon-Accra, Ghana; b Department of Materials Science & Metallurgy, University of Cambridge, 27 Charles Babbage Road, Cambridge, CB3 0FS, UK; c INCREASE (FR CNRS 3707), ENSIP, Université de Poitiers, 1 rue Marcel Doré, TSA41105, 86073 Poitiers Cedex 9, France; d Government Emerson College, Bahaudin Zakriya University, Multan, Pakistan

**Keywords:** Materials chemistry, Materials science, Engineering

## Abstract

Ag_3_PO_4_ photocatalyst has attracted interest of the scientific community in recent times due to its reported high efficiency for water oxidation and dye degradation. However, Ag_3_PO_4_ photo-corrodes if electron accepter such as AgNO_3_ is not used as scavenger. Synthesis of efficient Ag_3_PO_4_ followed by a simple protocol for regeneration of the photocatalyst is therefore a prerequisite for practical application. Herein, we present a facile method for the synthesis of a highly efficient Ag_3_PO_4_, whose photocatalytic efficiency was demonstrated using 3 different organic dyes: Methylene Blue (MB), Methyl orange (MO) and Rhodamine B (RhB) organic dyes for degradation tests. Approximately, 19 % of Ag_3_PO_4_ is converted to Ag^0^ after 4.30 hours of continuous UV-Vis irradiation in presence of MB organic dye. We have shown that the Ag/Ag_3_PO_4_ composite can be rejuvenated by a simple chemical oxidation step after several cycles of photocatalysis tests. At an optimal pH of 6.5, a mixture of cubic, rhombic dodecahedron, nanosphere and nanocrystals morphologies of the photocatalyst was formed. H_2_O_2_ served as the chemical oxidant to re-insert the surface metallic Ag into the Ag_3_PO_4_ photocatalyst but also as the agent that can control morphology of the regenerated as-prepared photocatalyst without the need for any other morphology controlling Agent (MCA). Surprisingly, the as- regenerated Ag_3_PO_4_ was found to have higher photocatalytic reactivity than the freshly made material and superior at least 17 times in comparison with the conventional Degussa TiO_2_, and some of TiO_2_ composites tested in this work.

## 1 Introduction

Photocatalysis is a promising technology that can be used as an important toolkit for addressing global energy crisis and address many environmental problems. It can be used for water and air treatment to destroy organic pollutants, toxic substances and bacteria. Compared to standard chemical processes, photocatalysis has the advantage of using sunlight to activate and drive degradation processes, and is therefore energetically sustainable and more eco-compatible. Since the photocatalytic splitting of water on TiO_2_ electrodes was reported in the early 1970's by Fujishima and Honda [1], there has been great interest in TiO_2_-based photocatalyst for renewable energy and environmental remediation applications.

TiO_2_ is reported to be efficient in degrading most organic dyes in the UV spectrum. TiO_2_ is primarily preferred because it is photoactive, biologically and chemically inert, photostable (i.e. not liable to photoanodic corrosion), inexpensive and non-toxic [2, 3, 4, 5]. However, it absorbs only in the UV spectrum. The UV region constitutes only 3–5 % of the sunlight compared to approximately 45 % for visible light. Therefore, the use of TiO_2_ photocatalyst for photocatalysis is not ideal for practical application. To achieve visible light active photocatalysis however, native, mixed metal, non-metal [6], sulphides [7] and nitrides [8] have been incorporated into pristine TiO_2_. However, efficiency of many of these photocatalysts have not been very encouraging and additional issues of stability of composites should be considered for some of the promising composites [9].

Since the pioneering work of Yi et al on Ag_3_PO_4_ for highly efficient photooxidation of water and dye degradation was demonstrated [10], there has been great interest in Ag_3_PO_4_ as a high quantum yield visible light active photocatalyst for oxygen evolution from water and for environmental remediation normally reported for dye degradation as the model system [11]. Its indirect and direct band gaps of 2.36 eV and 2.43 eV respectively, provide an appropriate transition for efficient absorption of visible light with wavelength shorter than 530 nm [12].

Recent research in Ag_3_PO_4_ has mostly focused on long term stability, surface area and tuneable facets synthesis [12, 13, 14]. Although, Ag_3_PO_4_, is highly efficient, stability remains an issue as it undergoes photo-corrosion upon irradiation. Particularly, the interstitial Ag^+^ in the crystal is reduced to Ag^0^ upon photo irradiation (A^+^ + e^−^ → A^0^). Therefore, the need to study Ag_3_PO_4_ with the view to understand and suppress photo-corrosion has attracted the interest of the scientific community in recent years. AgNO_3_ has been the preferred sacrificial scavenging agent [15] to prevent the photo-corrosion process. Considerable work has also been devoted to tunable size studies [16]. As particle size reduces, specific surface area and particularly, reactive sites also increase, again bulk recombination gets reduced with particle size reduction. However, if the feature size of say, a spherical particle is comparable with the electron mean free path, strong quantum confinement effect occurs, leading to high electron-hole pair recombination and presumably, reduction in efficiency [16]. However, investigation of optimal size for Ag_3_PO_4_ photocatalysis, sadly is still a scientific challenge. Additionally, since the presence of sharp edges, kinks, steps and defects on exposed facets result in high surface energy, high dye adsorption rate and arguably improved photocatalysis, facet-dependent photocatalytic activity of Ag_3_PO_4_ has also gained much attention. For example, organic dyes such as Rhodamine B (RhB), Methylene blue dye (MB) and Methyl Orange (MO) have been studied on different Ag_3_PO_4_ morphologies and different exposed facets. Particularly, trisoctahedral, rhombic dodecahedral, tetrahedral and cubic morphologies have shown better photocatalytic activity compared with pristine Ag_3_PO_4_ nanoparticles [17]. Comparing degradation efficiency of organic contaminants with different morphologies and exposed facets, Ag_3_PO_4_ single crystals with rhombic dodecahedrons having {110} facets showed higher catalytic activities than cubic {100} facets [14]. However, comparing photocatalytic production of oxygen on Ag_3_PO_4_ from oxidation of water on different facets and morphologies, (rhombic dodecahedrons composed of exposed {110} facets, cubic structures composed of exposed {100} facets and tetrahedral particles composed of exposed {111} facets) tetrahedral particles with {111} facets showed a 12 - fold increase [15]. However, to the best our knowledge, a simple protocol to rejuvenate Ag_3_PO_4_ photocatalyst chemically while simultaneously reproducing desirable with different morphologies and exposed facets after a continuous irradiation process is yet to be reported.

In this work, we present a facile method to synthesize a highly efficient Ag_3_PO_4_ composed of a mixture of cubic, rhombic dodecahedron, nanosphere and nanocrystals morphologies that can be demonstrated for degradation of Methylene Blue (MB), Methyl orange (MO) and Rhodamine B (RhB) organic dyes. The Ag_3_PO_4_ photocatalyst has much higher activity than the conventional Degussa TiO_2_, and some of its composite, and further can be recycled several times by chemically and rejuvenating after a cycle of irradiation treatment. H_2_O_2_ has been used to rejuvenate, maintain pH (6.5) of the reaction medium, control morphology and stabilise the regenerated Ag_3_PO_4_. The rejuvenated Ag_3_PO_4_ nanostructures retains its excellent photocatalytic activity, in fact improvement is shown from more desirable morphologies formed.

## 2 Experimental

All raw materials and chemicals in this work are of analytical grade and used without further purification. Powder samples were prepared by facile aqueous ion - exchange precipitation method [16, 18]. In a typical two - part synthesis procedure, “sample A” was prepared by completely dissolving AgNO_3_ (0.02 M [0.340 g]) in 100 ml distilled water under constant magnetic stirring. Aqueous solution of Na_2_HPO_4_ (0.02 M) was added dropwise to above solution and continuous stirred for 30 minutes. The yellowish products were collected via centrifugation and washed 3 times with deionised water. 3 ml H_2_O_2_ was added and then dried at 70 °C in darkness overnight.

“Sample B” was prepared by thoroughly mixing 1.41 g of Na_2_HPO_4_ and 1.6987 g of AgNO_3_ in an agate mortar and ground until the initial white colour changed to yellow. The mixture was washed with deionised water to remove any unreacted raw material; after which, 3 ml H_2_O_2_ was added and dried at 70 °C for 8 h in the dark. Finally, samples A and B were uniformly mixed to complete homogeneity in order to investigate the synergetic effect of the varied morphologies.

Powder X-ray diffraction (XRD) patterns of the Ag_3_PO_4_ composites were collected on a Bruker AXS - D8 Advance Bragg - Brentano diffractometer operating a copper tube (λ = 1.5418 Å) at 40 kV and 30 mA. The goniometer is equipped with a high-resolution setup (0.3° divergence slit, 2.5° incident and diffracted beam Soller slits, 6 mm receiving slit) and a curved-crystal graphite analyser, providing a narrow and symmetrical instrumental profile over the investigated angular range. The instrumental resolution function was characterised with the NIST SRM 660c (LaB_6_) standard [19, 20] all peak profiles were simultaneously fitted with symmetrical pseudo-Voigt functions whose width and shape were constrained according to the Caglioti et al formulae [21]. The XRD patterns of all specimens were recorded in the 10°–80° 2θ range with a step size of 0.0195° and a counting time of 20 s per step. Phase identification were made using the X'Pert HighScore Plus software. Raman spectroscopy measurements were carried out to confirm the structure, on a Raman spectrometer using a 633-nm laser excitation by raster scanning on average, 3μm steps over the samples with average exposure time of 60 seconds per pixel. The surface morphology of the as-prepared photocatalysts samples were carried out on a Field Emission gun FEI Nova NanoSEM scanning electron microscopy equipped with an EBSD- EDS acquisitions and operated at 30 kV. Samples were metalized with gold/platinum coating prior to the analysis. Images were acquired using a Gatan MiniCL imaging system at various magnifications. Absorption and diffused reflectance spectrophotometry (DRS) measurements were made on the samples using a USB - 4000 Ocean Optics UV−vis−NIR spectrophotometer equipped with a DRS probe. All measurements were made in air in the 200–800 nm range with a resolution of 1 nm. The chemical and elemental states of the samples were investigated on an ESCALAB 250 X-ray photoelectron spectrometer equipped with a monochromatized Mg KR X-ray source. The resulting binding energies were calibrated to the C1s (284.6 eV) peak.

The photo-induced degradation of organic dye was carried out with 50 mg Ag_3_PO_4_ powders suspended in a dye solution of MB (5 mg/L, 50 ml). The photocatalytic reactor included a quartz jacketed beaker equipped with a circulating jacket of cold water to ensure a steady temperature of 25 °C, a 150 W Xe lamp with optical filters (Newport BG40 for a close approximation of solar light and a 400-nm cut-off for visible light) and a magnetic stirrer with stir bar rotating at a speed of 100 rpm. Before illumination, the suspension was magnetically stirred in the dark for 30 minutes to achieve an adsorption/desorption equilibrium of dye on samples' surface. 2 ml aliquots were taken every minute and centrifuged at 3000 rpm for 10 minutes to remove the Nano powder. The concentration of dye was measured via absorbance values with an Ocean Optics UV-VIS USB 4000 spectrometer. Plastic 1.5 ml cuvettes were used with water calibration for absorbance measurements.

## 3 Results and discussion

**Fig. 1:**
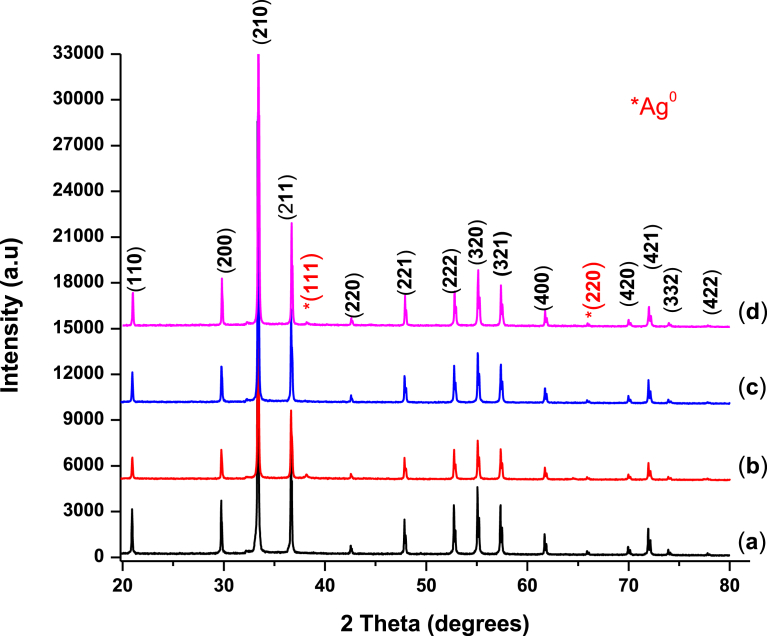
XRD patterns of Ag_3_PO_4_: (a) Pristine Ag_3_PO_4_; (b) Ag_3_PO_4_ after 4^th^ run of photocatalysis and (c) Rejuvenated Ag_3_PO_4_ and (d) Ag_3_PO_4_ after 4.30 hours of photocatalysis.

Ag_3_PO_4_ precipitates are yellow in colour and XRD patterns of our sample (Fig. 1 a) confirms this crystal structure. All patterns matched very well with the JCPDS card (1–84 - 192) standard data of Ag_3_PO_4._ Ag_3_PO_4_ forms a body - centred cubic BCC, type structure with P4 - 3n space group and lattice parameter of 6.004 Å [18]. The structure consists of isolated, regular PO_4_ tetrahedral (P – O distance of ∼ 1.539 Å) forming a body-centred cubic lattice. Six Ag^+^ ions are distributed among twelve sites of two - fold symmetry [10]. After 0.5–4.30 hrs of photocatalysis test with MB dye, new diffraction peaks are identified at 38.1° and 64.2° (Fig. 1 b and d). These are indexed as (111) and (220) crystal planes of metallic Ag (JCPDS card 1–1164) with average crystalline domain size of 16 nm (from WPPM). As can be seen, some amount of Ag^0^ is formed. However, upon rejuvenation, Ag^0^ is converted back to Ag^+^ (Fig. 1 c) within Ag_3_PO_4_. XRD patterns of rejuvenated Ag_3_PO_4_ is identical to the freshly made Ag_3_PO_4_. This indicates that the as-prepared Ag_3_PO_4_ can be recycled to the stable phase and thus a protocol for practical application can be developed on this basis.

**Fig. 2:**
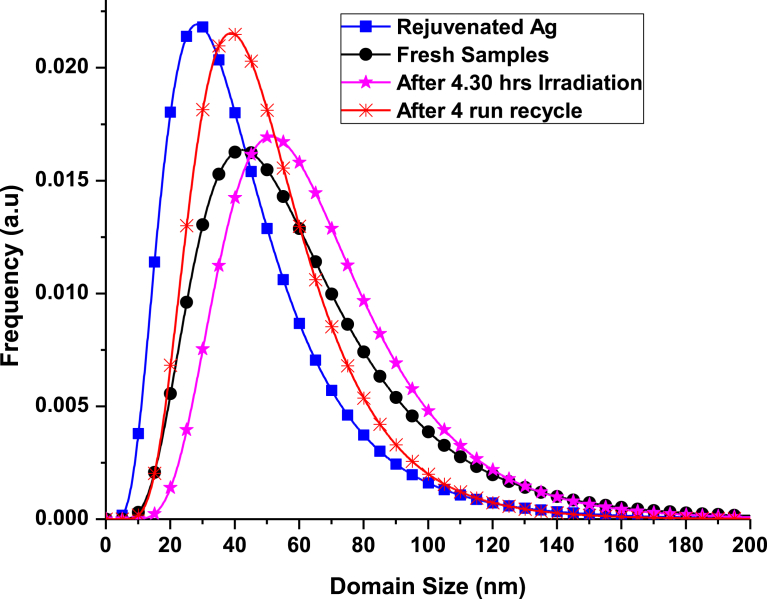
Crystallite domain size distribution.

Quantitative microstructural information was obtained from XRD data by means of the WPPM approach [22], a physically sound alternative to traditional line profile analysis based on the Scherer formula [23, 24, 25, 26]. WPPM directly connects a physical model for the microstructure with the diffraction pattern, allowing an extraction of microstructure parameters without recurring to arbitrary peak shapes to fit the diffraction peak profiles. The WPPM results obtained assuming the presence of a single phase Ag_3_PO_4_ with a lognormal distribution of cuboidal domains are presented in (cf. Table 1 and Fig. 2).

**Table 1:** Average domain size of the Ag_3_PO_4_.

Sample	Crystalline domain size
Fresh	62.2 nm
Rejuvenated	44.7 nm
4.30 hr	66.9 nm
After 4 cycles run	51.5 nm

**Fig. 3:**
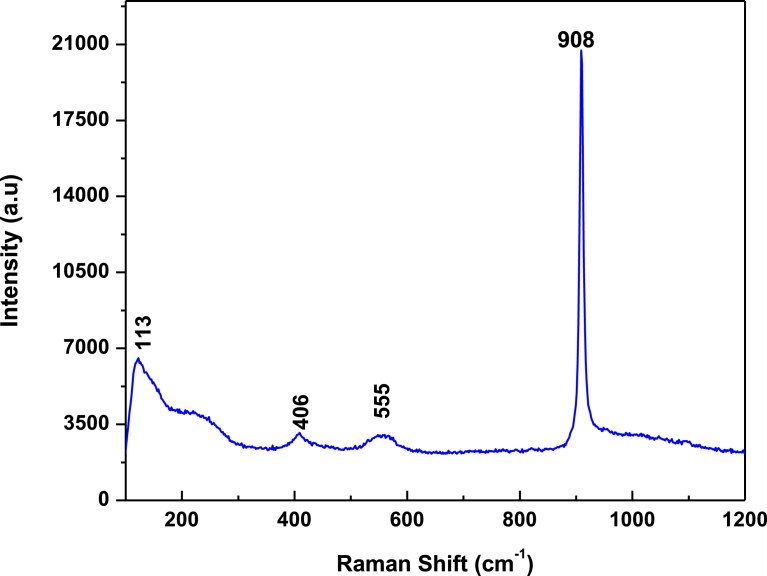
Raman spectra for pristine Ag_3_PO_4_ sample.

**Fig. 4:**
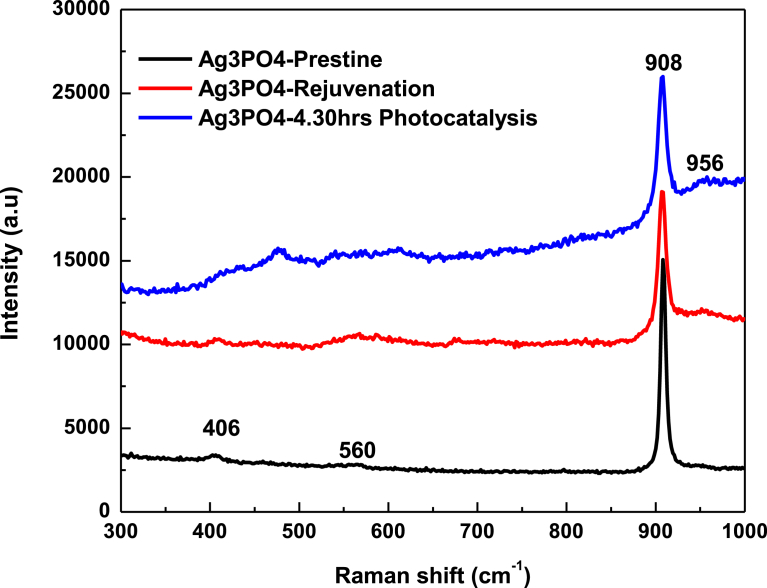
Raman spectra: Pristine Ag_3_PO_4_ before photocatalysis, rejuvenated Ag_3_PO_4_ sample and Ag_3_PO_4_ after 4.30 hr of photo catalysis respectively.

Raman spectra for pristine Ag_3_PO_4_ (Fig. 3); and Raman spectra for pristine Ag_3_PO_4_, rejuvenated Ag_3_PO_4_, and Ag_3_PO_4_ after 4.30 hr of photocatalysis (Fig. 4), confirm the formation of Ag_3_PO_4_ and its stability even after 4.30 hours of photocatalysis. The intense peak of 908 cm^−1^ is attributed to the terminal oxygen vibrational stretching of the PO_4_ group, whilst the very weak peak of 555 cm^−1^ associated to the asymmetric vibrational bending of the P-O-P bond, the weak peak of 406 cm^−1^ can be ascribed to the O vibrational bending of the PO_4_ and finally, the medium peak, 113 cm^−1^ attributed to the symmetry vibrational bending of Ag-O bonds [27, 28].

Bi et al [14] and Martin et al [15] have reported that tetrahedral morphology with {111} facets are ideal for O_2_ evolution from water whilst the rhombic dodecahedral with {110} facets favour dye degradation. Consequently, our goal was to synthesize a highly efficient but stable photocatalysts with mixed morphologies (mainly rhombic dodecahedral) that can be rejuvenated after Ag^+^ is reduced to Ag^0^ using wet chemical oxidation method. Wet chemical oxidation was first reported by Hue et al [29]. However, they produced mainly tetrahedral {111} morphology with rather low degradation rate. However, our rejuvenated Ag_3_PO_4_ can achieve high rate under 8 minutes for decomposing three different organic dyes. However, a direct comparison of different reported results must be treated with caution as differences may arise owing to experimental conditions such as photocatalyst loading, dye concentration, type of light source and intensity. Thus, we have compared standard commercially sourced TiO_2_ based photocatalytic materials under identical experimental conditions and evaluated rate constants for dye degradation.

**Fig. 5:**
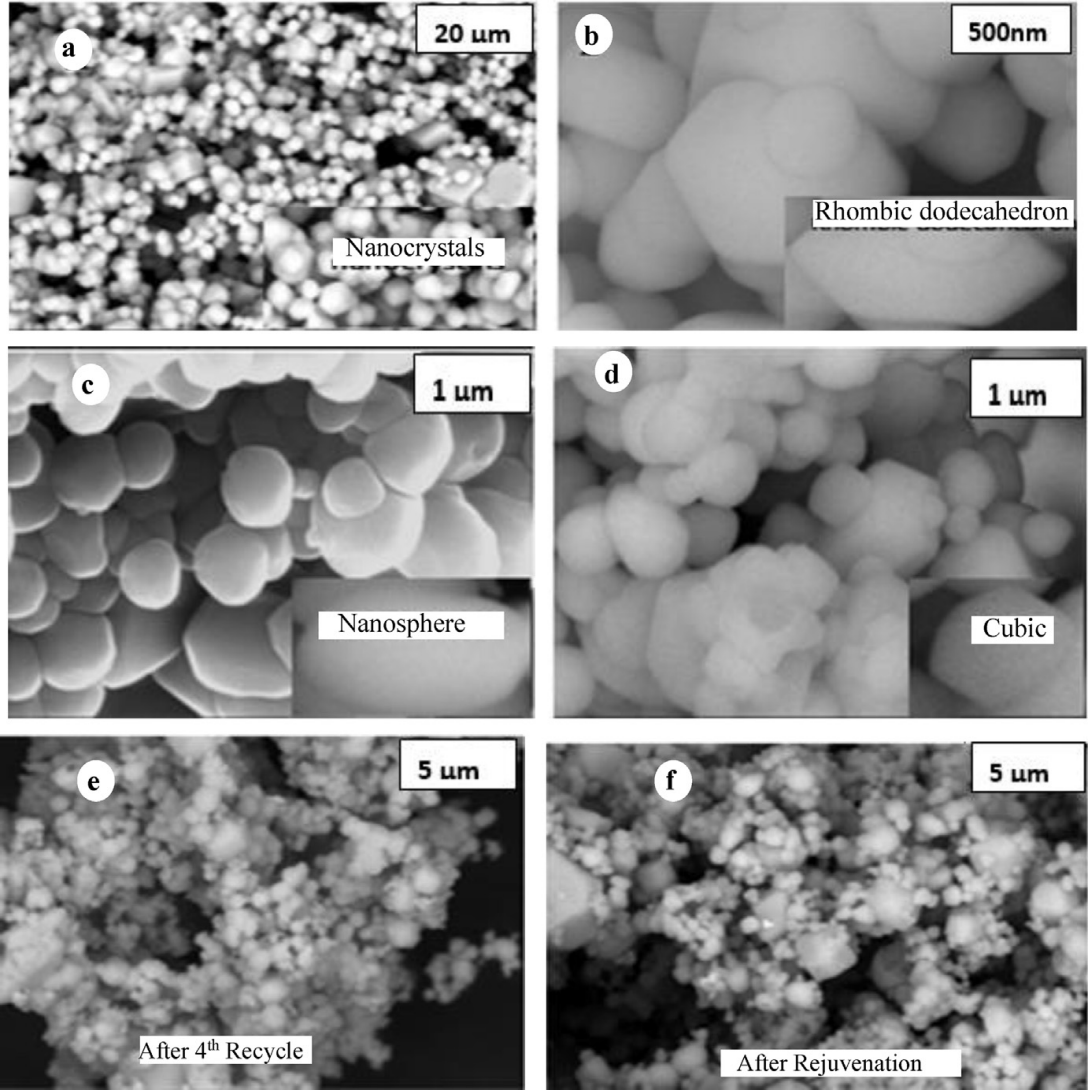
Scanning electron microscopy (SEM) images of the Ag_3_PO_4_ photocatalyst. (a) Nanocrystals; (b) Rhombic dodecahedral; (c) Nanosphere; (d) Cubic; (e) Ag_3_PO_4_ Photocatalyst after 4^th^ run of cycle; (f) Ag_3_PO_4_ Rejuvenation.

The Ag_3_PO_4_ photocatalyst after chemical rejuvenation with hydrogen peroxide is stable for re-use and remains highly efficient. H_2_O_2_ does not only act as oxidant but also as a morphology controlling agent (MCA) due to its pH variation ability which influences nucleation and growth. MCA such as, organic surfactants (e.g. PVP), or capping agents (e.g. fluorine ions) have been explored in the literature to synthesize Ag_3_PO_4_ of different morphologies of exposed facets [30, 31]. However, due to their strong interaction with substrates, complete removal of these MCAs has not been very successful. To avoid this problem, we have shown that the oxidant H_2_O_2_ itself can act as a MCA by achieving variations in pH to control the nucleation and growth such that different morphologies can be formed of the rejuvenated samples. It has been reported recently that if pH is maintained between a range of 6–9, cubic, tetrapods, trisoctahedrons, tetrahedrons and rhombic dodecahedral with mixed facets can be produced [15, 17], pH was therefore optimised to obtain required mixture of morphology. As Ag_3_PO_4_ can dissolve in strong acidic medium and H_2_O_2_ can easily decompose in strong alkaline medium, an optimal pH of 6.5 was maintained for achieving stable processing conditions. Under this pH nanostructures, in the form of nanocrystals, rhombic dodecahedron, nanosphere and cubes with average size < 150 nm were formed. Presence of these morphologies have been confirmed by SEM micrographs in Fig. 5 (a–d). After the 4^th^ run of dye degradation, formation of metallic Ag is confirmed by both XRD and SEM. It is known that Ag_3_PO_4_ is photosensitive whereby, some interstitial Ag^+^ ions can be converted to metallic Ag^0^, forming mostly nanocrystals of the metal on the surface of the photo catalyst (Fig. 5e). In small concentrations, Ag in contact with the photocatalyst can serve as an electron acceptor thus reducing e-h recombination and promoting hole reactivity. In large concentrations, Ag can block the surface of the photocatalyst thus decreasing photo activity. Thus, after the activity is decreased, it is important to regenerate the photo-activity of the photocatalyst.

Upon rejuvenation, relatively more rhombic dodecahedron and cubic morphologies with {110} and {100} facets respectively are formed compared to that after 4th cycle test samples (Fig. 5f). Since it is difficult to determine the crystal facet by TEM observation, owing to Ag_3_PO_4_ being photosensitive and prone to degradation, XRD patterns can be used to predict facets of the as-prepared samples [17]. Exposed crystal facets of reported Ag_3_PO_4_ characterized by {110} and {100} are identified as rhombic dodecahedral and cubic respectively, therefore, it is reasonable to conclude that the as-prepared Ag_3_PO_4_ would preferentially attach along the same orientation, leading to a similar nucleation and growth processes. Interestingly, the visual image morphologies of the rejuvenated Ag_3_PO_4_ is mostly like the as-prepared (fresh) samples. This therefore confirms the hypothesis that the fresh silver orthophosphate photocatalyst can be rejuvenated after several series of photocatalytic activity cycles or irradiation time.

**Fig. 6:**
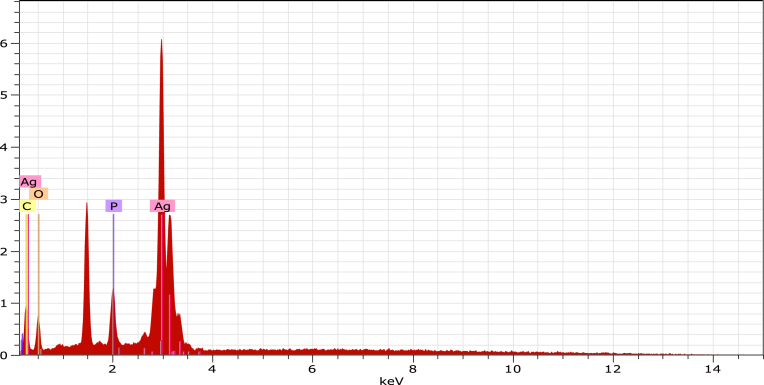
EDS analysis of the Ag_3_PO_4_.

The composition of the photocatalyst was determined by energy dispersive spectroscopy EDS (Fig. 6). The EDS spectrum confirmed elements of Ag, P, O, and C. The presence of Pd impurities can be attributed to Pd coating used to avoid surface charging during sample preparations.

**Fig. 7:**
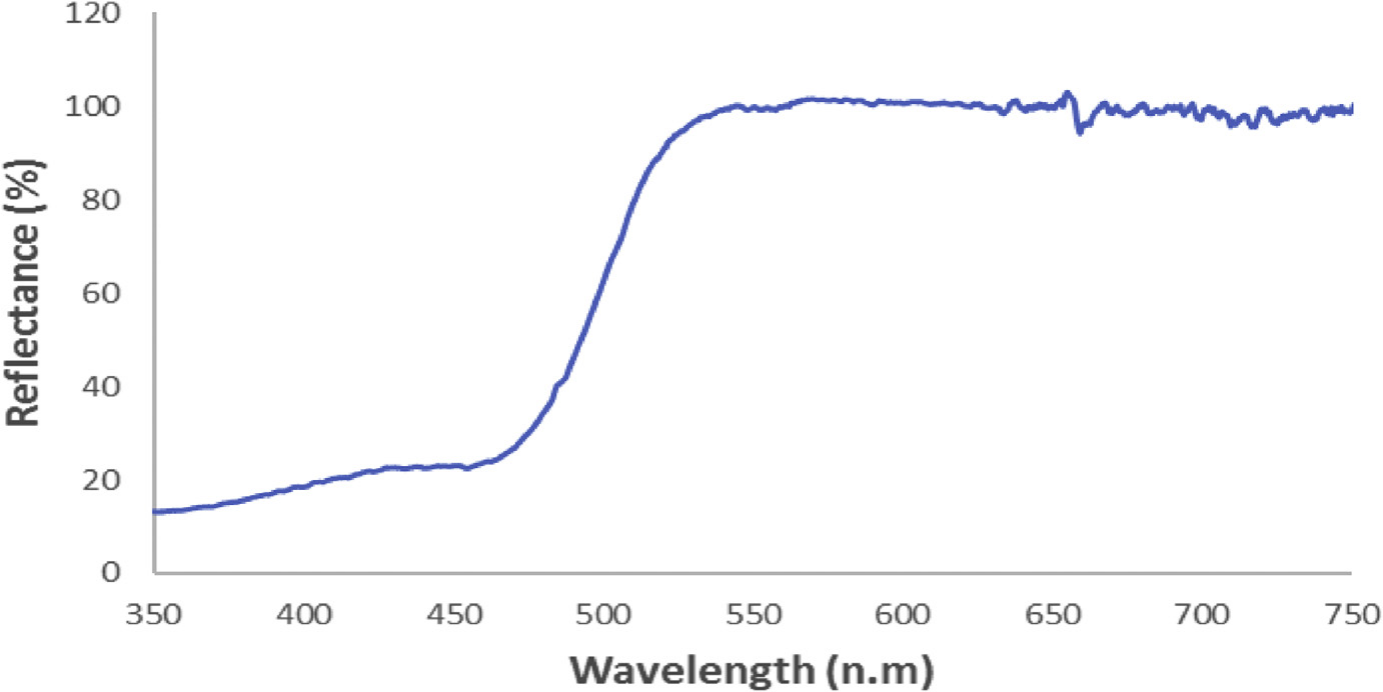
UV-Vis diffuse reflectance spectra of Ag_3_PO_4_.

Fig. 7 shows the diffuse reflectance spectra of Ag_3_PO_4_ samples. As shown in Fig. 7, the range of most intense light absorption of the Ag_3_PO_4_ photocatalyst occurs for wavelengths <530 nm. This is characteristic of Ag_3_PO_4_ band edge and it is attributed to the indirect band gap of 2.36 eV for Ag_3_PO_4_ [10].

**Fig. 8:**
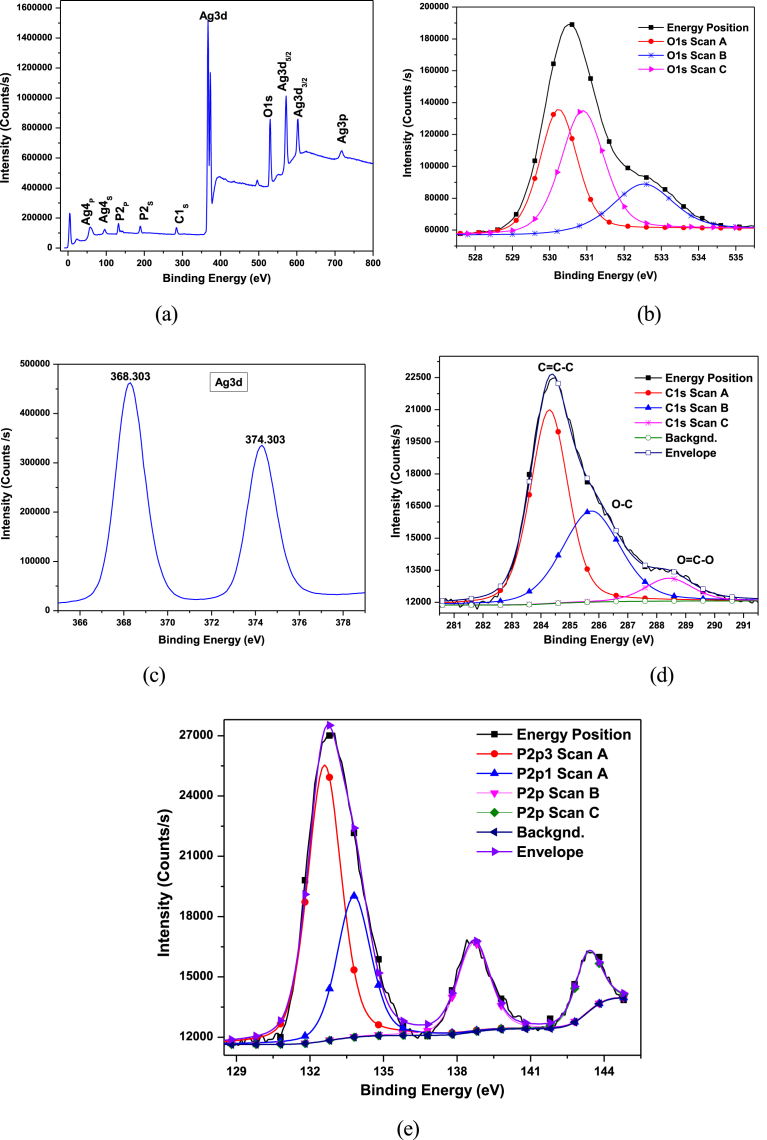
X-ray photoelectron spectra of the Ag_3_PO_4_ photocatalyst: (a) Survey XPS spectra of as prepared Ag_3_PO_4_; (b) High-resolution XPS spectra of O1s, (c) High-resolution XPS spectra of Ag3d; (d) High-resolution XPS spectra of C1s; (e) High-resolution XPS spectra of P 2p.

X-ray photoelectron spectroscopy (XPS) of elemental composition, chemical status, and silver content of as-prepared Ag_3_PO_4_ were further analysed. The Ag_3_PO_4_ samples contain the elements Ag, O, P and C, with binding energies (eV) located at 368.3 eV (Ag 3d), 530.2 eV (O 1s), 132.4 eV (P 2p), and 284. 5 eV (C 1s) respectively (Fig. 8 a–e).

It is observed that the peaks of Ag 3d_5/2_ and Ag 3d_3/2_ are located at 368 eV and 374 eV, respectively. The Ag 3d_3/2_ and Ag 3d_5/2_ peaks are further divided into two different peaks: 374.6 eV and 374.08 eV; and 368.6, 368.05 eV respectively. The 374.08 eV and 368.05 eV are attributable to Ag^+^ ions (in Ag_3_PO_4_) [18]. Adventitious carbon contamination peaks with binding energies of 284.8 eV, ∼286 eV and ∼288.5 eV correspond to the C-C, C-O and O-C=O bonds, respectively. The results of the XPS, corroborate both XRD and Raman results.

Reaction between the precipitating agent (Na_2_HPO_4_) and the AgNO_3_ precursor, produced three products: The Ag_3_PO_4_ photocatalyst, aqueous sodium nitrate and nitric acid as described in Eqn. (1). The photocatalyst precipitates out as a sparingly soluble salt with a solubility product of 8.89 × 10^−17^ at 25 °C. However, since sodium nitrate easily dissolves in water (solubility of approximately, 2.570 kg/litre at room temperature), the synergistic effect of the oxidant, sodium nitrate and nitric acid provide an optimised pH of 6.5.

(1) $$3AgNO_{3}+Na_{2}HPO_{4}\overset{H_{2}O_{2\;}\left(oxidant\right)}{\to }Ag_{3}PO_{4↓}+2NaNO_{3}+HNO_{3}$$

Since Ag_3_PO_4_ photo corrodes with time, stability of silver orthophosphate photocatalyst for practical application is important. To this end, experiments for rejuvenation of Ag^0^ to Ag^+^ after series of dye degradation tests was performed.

**Fig. 9:**
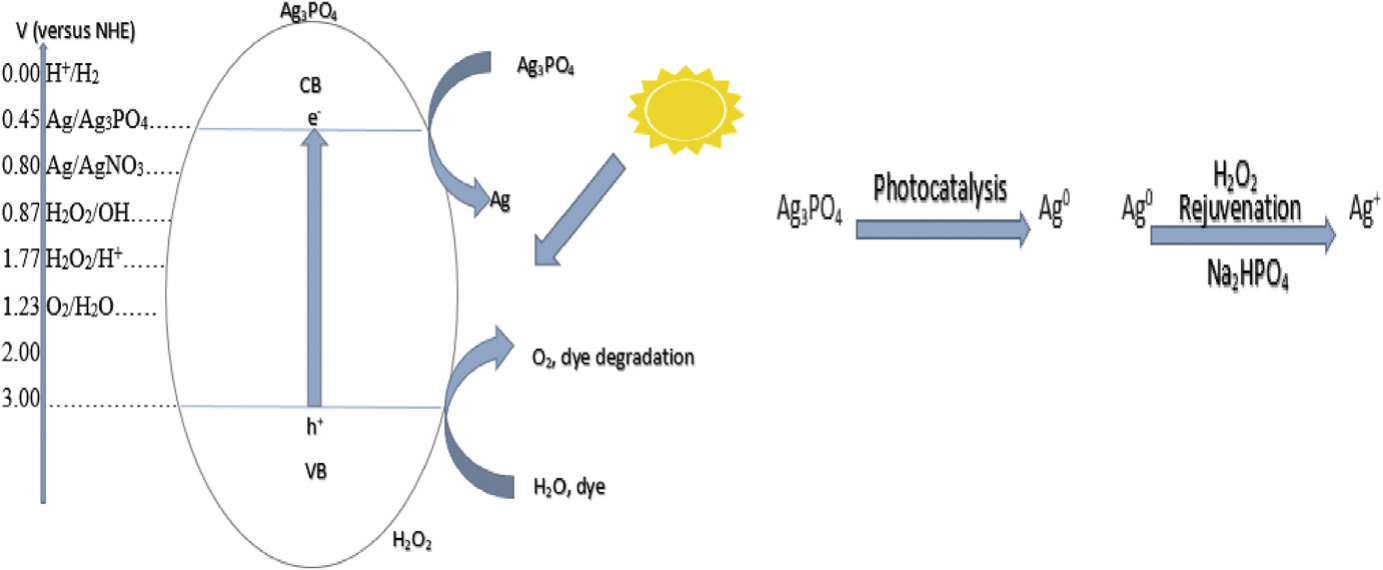
Redox potentials of Ag_3_PO_4_ (left) and reversible photocatalytic activity of Ag_3_PO_4_ (right).

Fig. 9 illustrates that the conduction band electrode potential of the photocatalyst of +0.45 V is more positive than the Normal Hydrogen Electrode (NHE), relative to the reduction potential of 0.00 V for hydrogen proton (H^+^) standard reduction potential. Consequently, there is a driving force for Ag^+^ to photo-corrode to Ag^0^ by the photogenerated electrons (to form Ag/Ag_3_PO_4_) if a scavenging agent such as AgNO_3_ in the solution is not used [29]. Eqn. (2) shows the reaction mechanism through which Ag_3_PO_4_ decomposes to Ag^0^:

(2) $$Ag_{3}PO_{4}+3e\;\to 3Ag+2HPO_{4}^{3-}$$

Upon visible light irradiation with wavelength less than 530 nm (eV > Eg), the electron-hole pairs generated from Ag_3_PO_4_ are separated and electrons at the conduction band edge reduce Ag_3_PO_4_ to Ag^0^ (**A**^**+**^ **+ e**^**−**^ **→ A**^**0**^). The hole at the valence band can directly oxidize organic dye or the adsorbed water molecules to form hydroxyl radicals and hydrogen peroxide (Fig. 9). However, the weakly elemental Ag can be rejuvenated by using hydrogen peroxide as an oxidant and a weaker alkaline (Na_2_HPO_4_) as PO_4_^3**−**^ ions source according to the reaction in Eqn. (3) [29].

(3) $$6Ag+3H_{2}O_{2}+2HPO_{4}^{2-}\to 2Ag_{3}PO_{4}+2H_{2}O+4OH^{-}$$

**Fig. 10:**
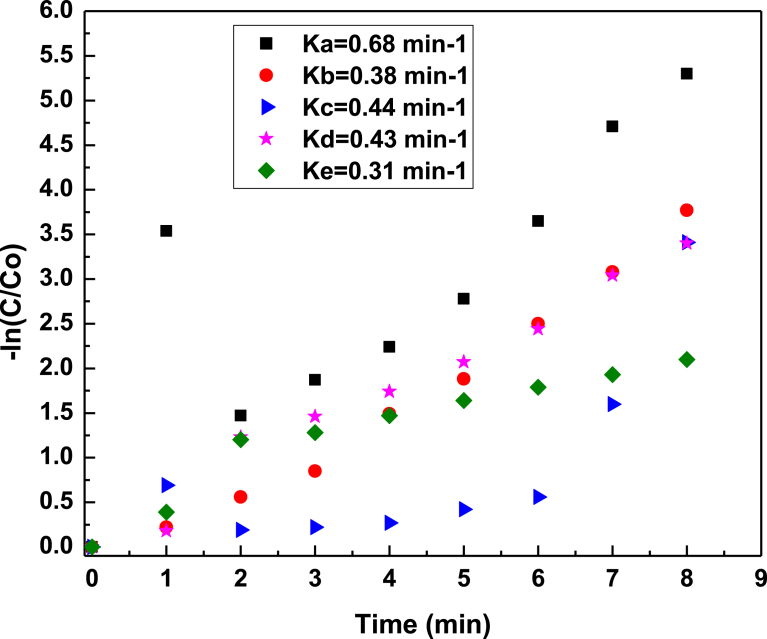
Kinetic curves for dye degradation: (Ka) MB degradation with rejuvenated; (Kb) MO degradation with fresh Ag_3_PO_4_; (Kc) RhB degradation with fresh Ag_3_PO_4_; (Kd) MB degradation with fresh Ag_3_PO_4_; (Ke) MB degradation with 4th recycled Ag_3_PO_4_.

**Fig. 11:**
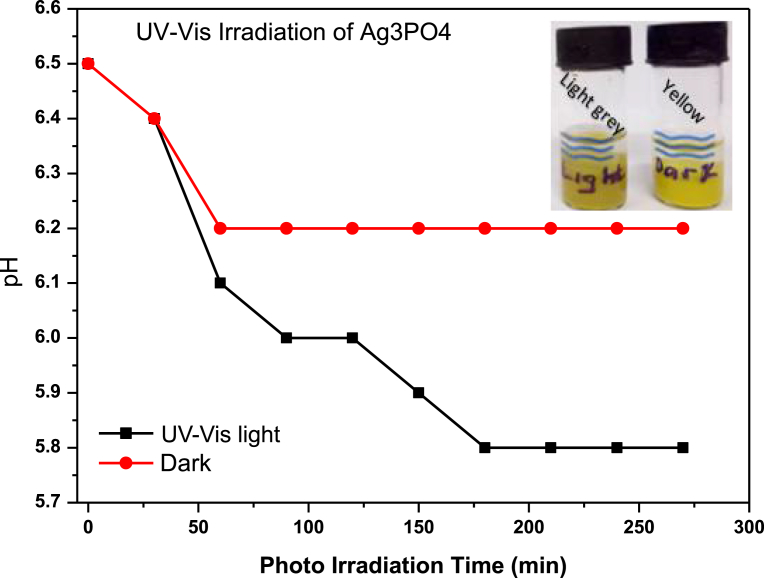
Variation of pH with irradiation time. Insert shows Ag_3_PO_4_ irradiated with UV-vis light and non-irradiation respectively. Irradiated Ag_3_PO_4_ shows light grey colour, an indication of partial Ag^0^ formation whilst non-irradiated shows yellow, indicating a stable Ag_3_PO_4_.

It has been reported by Liu et al [18] that the conversion of Ag^+^ to Ag^0^ under visible light irradiation, follows colour change from yellow, light grey, grey, dark grey and dark after 0.5 hr, 1 hr, 1.5 hr, 2 hr and 3 hr respectively. After multiple cycles of photocatalysis, Ag^0^ precipitate as a film and as nanocrystals around the surface of Ag_3_PO_4_, forming Ag^0^/Ag_3_PO_4_ composite. The pH of 6.5 is altered with subsequent decrease in photocatalysis efficiency, therefore knowledge of Ag^+^ to Ag^0^ conversion rate and its relation to pH of the photocatalyst was observed to be crucial for predicting stability of the as-prepared Ag_3_PO_4_. Fig. 11, shows relationship between pH and irradiation time. The pH of irradiated Ag_3_PO_4_ changed from 6.5 to 5.8. In contrast, the non-irradiated Ag_3_PO_4_ changed from 6.5 to 6.2. The latter observation, can be attributed to marginal degradation from background light.

**Fig. 12:**
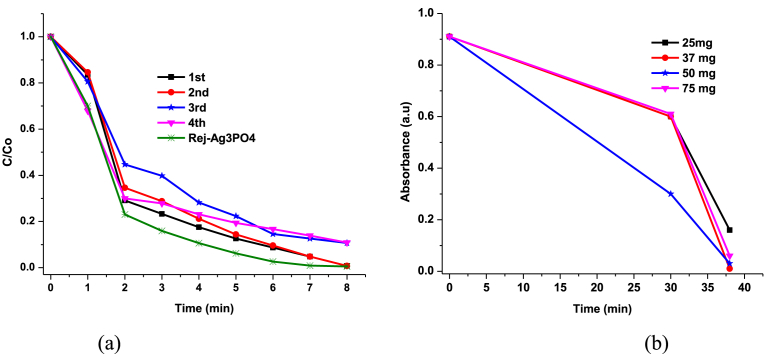
(a) Efficiency of Ag_3_PO_4_ with evolution of time; (b) Optimisation of photocatalyst loading.

**Fig. 13:**
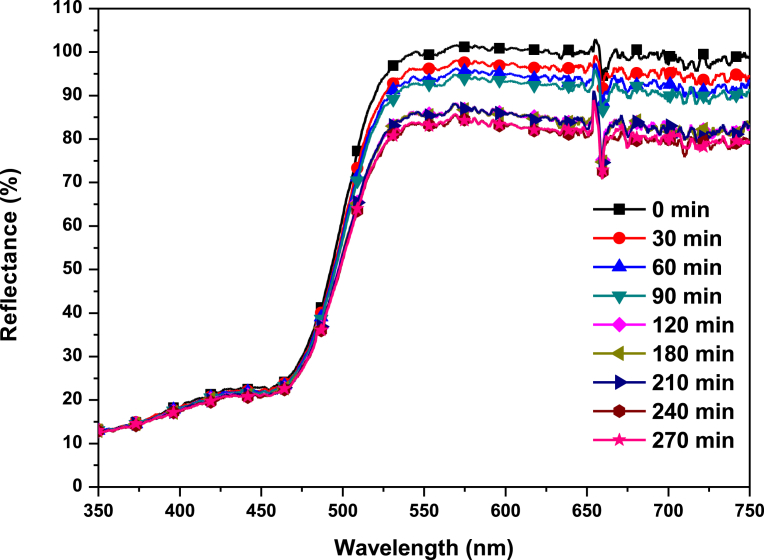
Diffuse Reflectance of Ag_3_PO_4_ under UV- visible light irradiation.

It is interesting to note that after 4.30 hours of irradiation, colour changed from yellow to only light grey (Fig. 11, insert). This suggests that pH can be controlled between 6.5–5.8 under 4.30 hrs irradiation to form Ag^0^/Ag_3_PO_4_ composite. This composite can utilise the surface plasmonic resonance effect of the Ag nanoparticles to improve the overall efficiency of the photocatalyst. However, efficiency of 4^th^ run (Fig. 12 (a)), reduced by 10 % presumably due to low adsorption rate on active sites of the photocatalyst as the Ag^0^ film can occlude dyes from directly adsorbing onto the photocatalyst. It can be inferred again that approximately, 19 % Ag^+^ is converted to Ag^0^. To further probe this hypothesis, the photocatalyst was irradiated for 4.30 hrs and diffuse reflectance taken every 30 minutes. Fig. 13, shows the reflectance (%) of the as-prepared Ag_3_PO_4_ photocatalyst subjected to UV-Visible light irradiation.

**Fig. 14:**
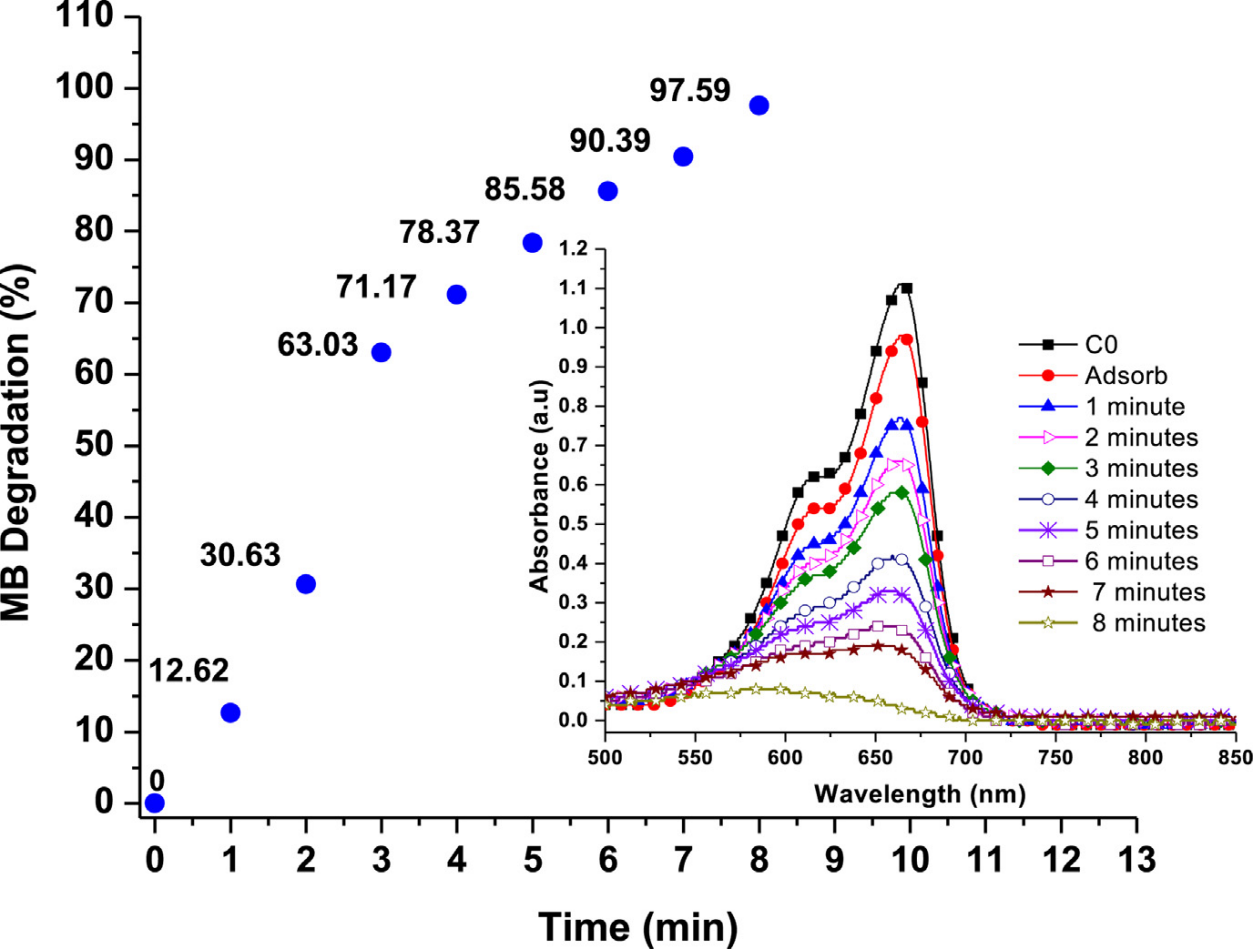
Percentage degradation of MB catalysed by Ag_3_PO_4_ under visible light irradiation (insert: Temporal evolution of spectra change for the MB dye).

It is interesting to note that, reflectance decreased from 100 % (at 0 minute) to approximately 80.7 % after 4.30 hrs of irradiation, consistent with the colorimetric observation (Fig. 11). Photocatalysis of the 4.30 hrs irradiated Ag_3_PO_4_ degraded 97 % of MB under 8 minutes in visible light irradiation (Fig. 14).

**Fig. 15:**
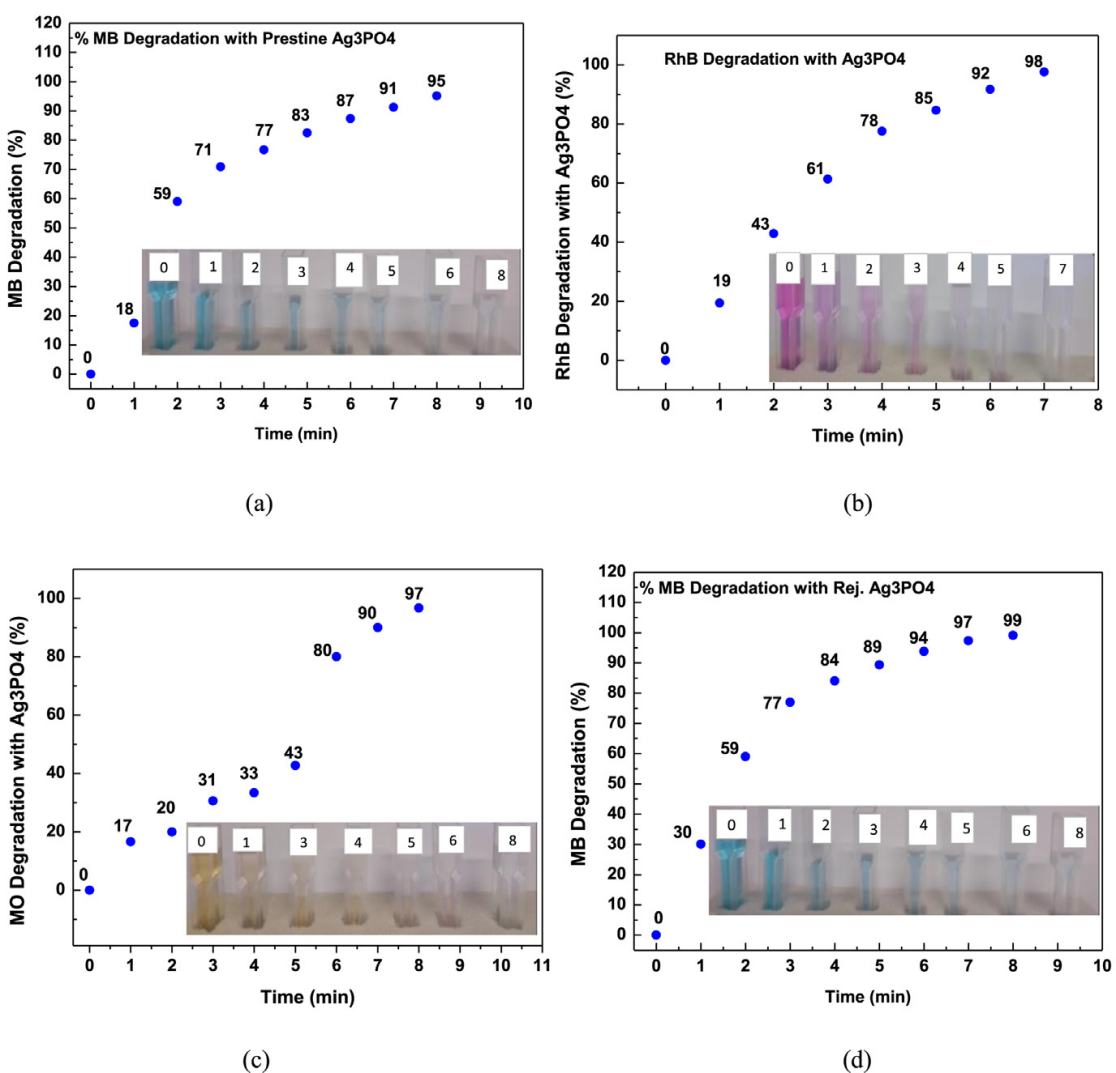
Percentage degradation of molecular dyes with time. (a) MB degradation with fresh Ag_3_PO_4_; (b) RhB degradation with Ag_3_PO_4_; (c) MO degradation with fresh Ag_3_PO_4_ and (d) MB degradation with Rejuvenated Ag_3_PO_4_. (Inserts: colour change for photocatalysis of MB, RhB, MO and MB respectively).

We must however, point out that in these irradiation experiments, catalysts were irradiated in the absence of dyes, thus accounting for the relatively high efficiency compared to the 4^th^ run test sample (Fig. 15). Overall, the photocatalyst was stable and efficient and would be ideal for practical photocatalysis application since it can be continuously run for more than 4.30 hours before the need for rejuvenation. Secondly, a facile, low temperature and pH controllable photocatalyst for achieving tailored morphologies and Ag^0^/Ag_3_PO_4_ composite has been proposed.

**Fig. 16:**
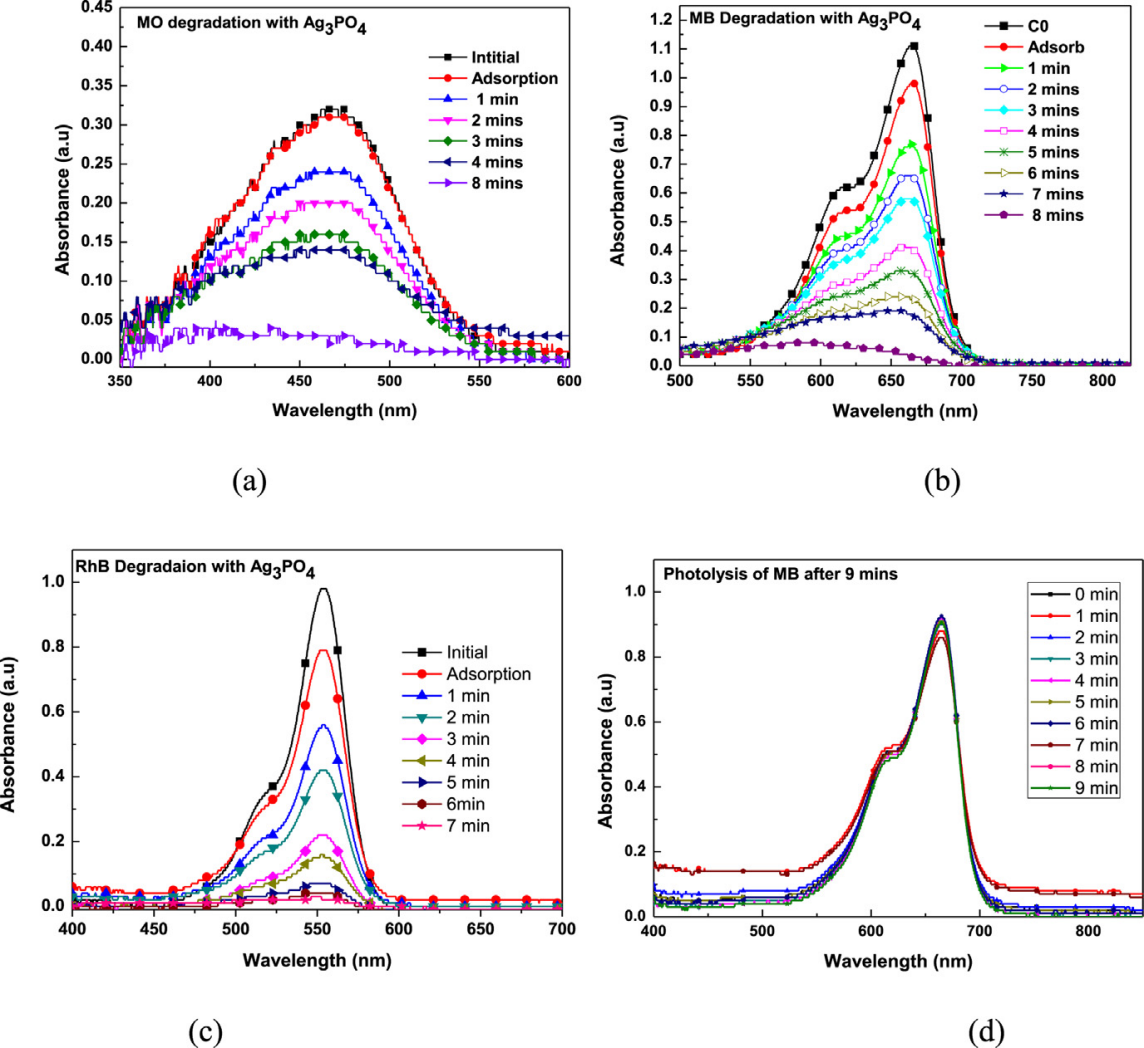
Temporal evolution of spectra change for the dyes: (a) Methyl Orange degradation; (b) Methylene Blue degradation; (c) Rhodamine B degradation and (d) Methylene blue photolysis.

The photocatalytic activity of the as-prepared sample was evaluated by determining the degradation rate of Methyl orange, Methylene blue and Rhodamine B dyes (Fig. 16 (a–c)). Temporal evolution of spectra change for the three dyes occurs at 462 nm, 665 nm and 550 nm respectively. It can be observed that there is a successive decrease in absorbance maximum with increasing irradiation time. However, in the absence of Ag_3_PO_4_, absorbance maximum remains the same (Fig. 16 d).

This indicates that degradation of dyes was mainly due to the presence of the Ag_3_PO_4_ photocatalysts. Complete dye degradation occurred after 7 minutes for RhB and 8 minutes for both MO and MB. Though the chromophore in each experiment degrade completely, it does not mean complete mineralisation of dyes into inorganic CO_2_ and H_2_O also occur. Fig. 15 (a–c), shows percentage degradation with time. The highest percentage of 97.66% was achieved for RhB under 7 minutes (Fig. 15 b). It was followed by 96.7% for MO (Fig. 15 c) and 95.20% for MB (Fig. 15 a) respectively.

For water purification and oil spill purposes, it would be interesting to determine the rate of mineralisation of organic pollutants since intermediate products may be more hazardous than their pristine forms.

The conversion of Ag^+^ to Ag^0^ metal, results in reduced efficiency. Hence, photocatalytic activity decreases with repeated cycles (Fig. 12 a). Efficiency reduced to approximately 10 % after the 4^th^ run of test. However, if the weakly elemental Ag^0^ is rejuvenated, a highly efficient Ag_3_PO_4_ is generated. Interestingly, when the photocatalyst was rejuvenated, the efficiency was 99.1% (Fig. 15 d), even higher than the fresh Ag_3_PO_4_ photocatalysts. This result can be explained as follows: during rejuvenation, the optimal pH (6.5) was achieved. Hence, more rhombic dodecahedral and cubic morphologies are formed for rejuvenated samples compared to recycled samples. These morphologies with {110} and {100} facets provide more sharp edges, corners and high surface energy. Additionally, the presence of both nanocrystals and fine nanoparticles provide relatively large specific surface area. The synergistic effect of the mixed morphologies of rejuvenated rhombic dodecahedral, cubic, nanosphere and nanocrystals accounts for the superior photocatalytic performance of the rejuvenated Ag_3_PO_4_.

To optimize parameters for photocatalysis, factors such as light intensity and dye concentration, photocatalyst loading and type of light source among others, are very important. Consequently, the optimal Ag_3_PO_4_ loading was determined, as shown in Fig. 12 b, at 50 mg loading, absorbance peak (665 nm) of methylene blue completely disappeared after 8 minutes of irradiation. Photocatalyst loading of 50 mg was therefore the optimal loading used throughout the experiments.

**Fig. 17:**
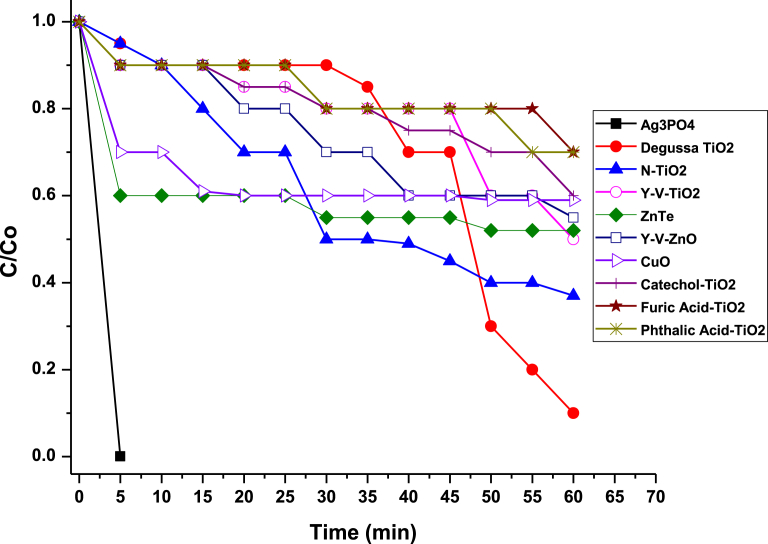
Comparison of the efficiency of Ag_3_PO_4_ photocatalysts with other common photocatalysts.

To utilise photocatalysis to solve practical environmental problems, photocatalysts must be designed to harness most of the visible light of the electromagnetic spectrum. Our effort was geared toward achieving this aim. Hence, TiO_2_ modified photocatalysts such as metal and non-metallic doped, other metal oxide and metal-organic frameworks (MOFs) were used as bench mark. However, as shown in Fig. 17, Ag_3_PO_4_ out performed them all. Under UV-vis irradiation, 99.11 % of MB degradation was achieved under 5 minutes compared to 90 % for Degussa TiO_2_ under 60 minutes. The Ag_3_PO_4_ is not only efficient but also stable and can be photo irradiated for more than 4.30 hours before rejuvenation. Considering this fact, we can speculate that this high efficient photocatalyst would make up for cost for intermittent rejuvenation especially when compared to conventional high capital-intensive remediation methods, particularly in oil spill remediation efforts.

To quantify the photocatalytic activities, the rate constants (k) of the dye for photocatalysis with fresh, repeated cycled and rejuvenated photocatalyst were determined.

From the kinetic curves in Fig. 10, the –ln(C/C_0_) generally varies linearly with reaction time, therefore the photodegradation can be treated as a first order reaction and the rate constant k, obtained from the linear simulations. The apparent rate constant obtained for each catalyst is summarised in Table 2.

**Table 2:** Kinetic constants for MB, MO and RhB dyes degradation catalysed by as-prepared Ag_3_PO_4_ and Degussa TiO_2_.

Photocatalysts	Organic dyes	Rate constant (−ln[C/Co])/min
Rej. Ag_3_PO_4_	MB	KA = 0.68
Fresh Ag_3_PO_4_	MO	KA = 0.38
Fresh Ag_3_PO_4_	RhB	KA = 0.44
Fresh Ag_3_PO_4_	MB	KA = 0.43
4^th^ Run Cycled Ag_3_PO_4_	MB	KA = 0.31
Degussa TiO_2_	MB	KA = 0.04

The highest (k) for photocatalytic activity (0.68 min^−1^) was achieved for the rejuvenated Ag_3_PO_4_. The lowest activity (0.04 min^−1^) was obtained for Degussa TiO_2_. The rate of improvement was about 17 times compared to the commercially available Degussa TiO_2_ and a dozen times better than the rest of the photocatalysts. It should be noted that the higher photocatalytic reactivity of the rejuvenated Ag_3_PO_4_ is possibly due to the synergistic effect of high surface area and presence of corners and sharp edges in the rejuvenated samples.

**Fig. 18:**
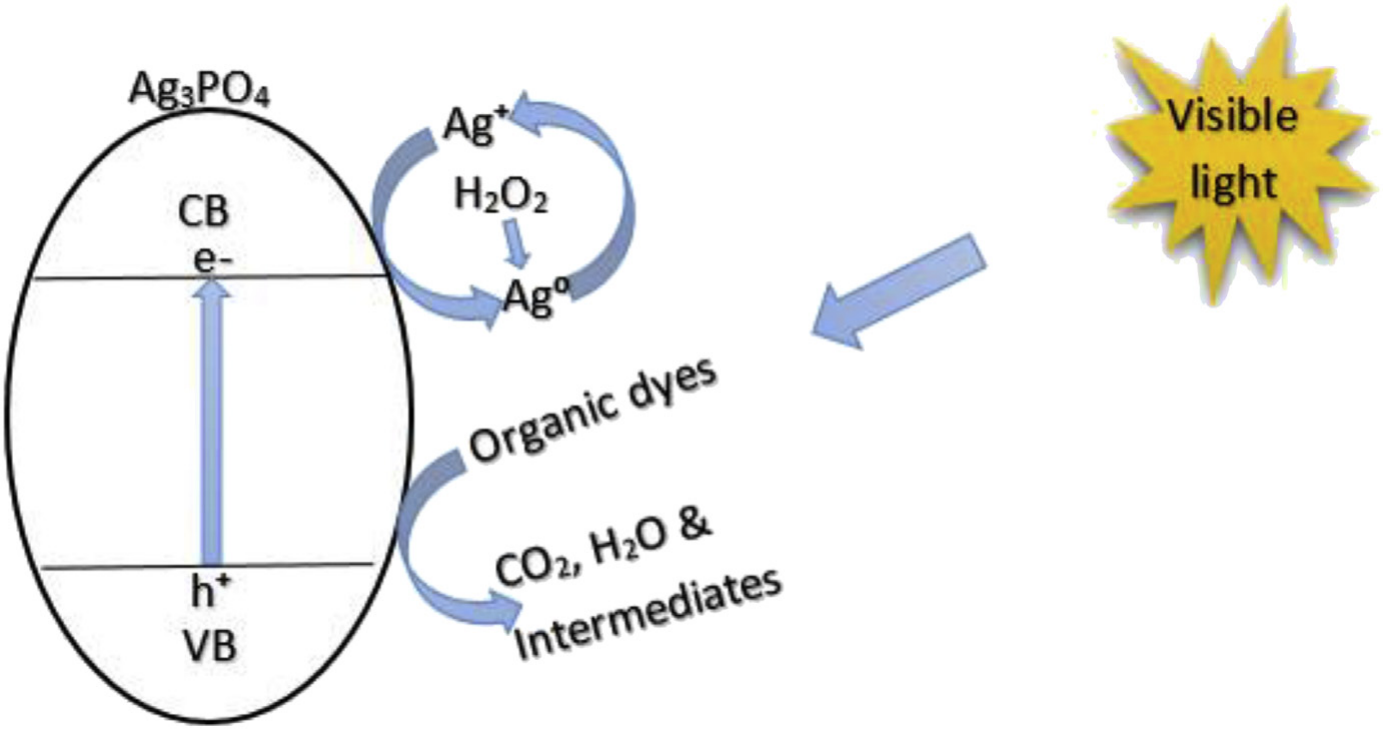
A schematic for mechanism of photocatalysis of Ag_3_PO_4_.

To explore predominant active species responsible for the high efficiency of the Ag_3_PO_4_, scavenging experiments were performed. The reactive oxygen species experiments were conducted as previously described [32]. Three typical chemicals: Ethylene diamine tetra acetic acid disodium (EDTA-2Na), 2,3-bis-(2-methoxy-4-nitro-5-sulfophenyl)-2-H-tetrazolium-5-carboxanilide (XTT) and coumarin were used as scavengers for detecting hole (h^+^ [33]), superoxide radicals (•O_2_^**−**^ [34]), and (•OH^−^ [35]) respectively. The likely mechanism is described as follows (Fig. 18), upon photon irradiation, photo-reduced Ag_3_PO_4_ (at the conduction band) is subsequently oxidised into Ag_3_PO_4_, with H_2_O_2_ as oxidant. Whilst the photo-excited hole (h^+^), at the valence band, directly oxidises MB, MO and RhB dyes into carbon dioxide, water and intermediate products.

**Fig. 19:**
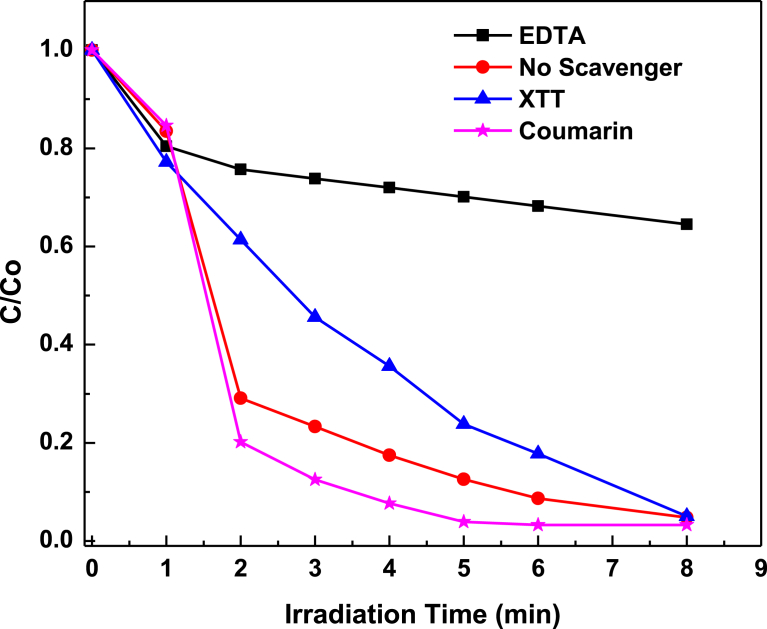
Scavenging experiments for probing the presence of hole (h^+^), superoxide radicals (•O_2_^**−**^), and (•OH^−^).

As depicted in Fig. 19, when 1 mM EDTA was used as scavenger in the photocatalysis experiments, degradation efficiency was greatly suppressed. The efficiency decreased from 99.11 % to 35.51 %. When same concentrations were used for both XTT and Coumarin, their efficiencies were 95.02 % and 96.74 % respectively. These suggest that the high efficiency of Ag_3_PO_4_ was mainly due to the presence of the hole (h^+^), with a high oxidation potential of + 2.45V vs NHE, like that observed by Yi et al [10].

## 4 Conclusions

In summary, a facile method to synthesize an efficient and stable Ag_3_PO_4_, composed of cubic, rhombic dodecahedron, nano spheres and nanocrystals morphologies for degradation of MB, MO and RhB has been demonstrated. By controlling pH at 6.5, controlled morphology can be produced. Approximately, 19 % of Ag^+^ is converted to Ag^0^. The Ag_3_PO_4_ photocatalyst can be rejuvenated after employing for more than 4.30 hours of irradiation. Both the fresh and the rejuvenated Ag_3_PO_4_ nanostructures have higher photocatalytic reactivity than conventional Degussa TiO_2_, and some modified TiO_2_. Ag_3_PO_4_ was found to be efficient and stable even after repeated cycles. It is hoped that this work would inspire exploration of similar method to control the morphology and stability of other easily photo-corroded photocatalyst for efficient water purification, oil spill and general industrial waste water treatment.

## Declarations

### Author contribution statement

Henry Agbe: Conceived and designed the experiments; Performed the experiments; Analyzed and interpreted the data; Wrote the paper.

Nadeem Raza: Conceived and designed the experiments; Analyzed and interpreted the data.

Aditya Chauhan, David Dodoo-Arhin and Vasant Kumar: Analyzed and interpreted the data.

### Funding statement

This work was supported by University of Ghana/University of Cambridge Commonwealth split-site PhD programme.

### Competing interest statement

The authors declare no conflict of interest.

### Additional information

No additional information is available for this paper.
